# Sperm Storage and Use Following Multiple Insemination in *Aedes albopictus:* Encouraging Insights for the Sterile Insect Technique

**DOI:** 10.3390/insects15090721

**Published:** 2024-09-20

**Authors:** Hanano Yamada, Rebecca Hood-Nowotny, Christian Resch, Jeremy Bouyer, Roman Gruber, Clelia F. Oliva

**Affiliations:** 1 Insect Pest Control Laboratory, Joint FAO/IAEA Centre of Nuclear Techniques in Food and Agriculture, International Atomic Energy Agency, Vienna International Centre, P.O. Box 100, 1400 Vienna, Austria; jeremy.bouyer@cirad.fr; 2 Department of Forest and Soil Sciences, Institute of Soil Research, University of Natural Resources and Life Sciences Vienna, Konrad-Lorenz-Strasse 24, 3430 Tulln, Austria; rebecca.hood@boku.ac.at; 3 Soil and Water Management and Crop Nutrition Laboratory, Joint FAO/IAEA Centre of Nuclear Techniques in Food and Agriculture, International Atomic Energy Agency, Vienna International Centre, P.O. Box 100, 1400 Vienna, Austria; ch.resch@iaea.org (C.R.); romanchemie@gmail.com (R.G.); 4 ASTRE, CIRAD, INRAE, University of Montpellier, Plateforme Technologique CYROI, 97490 Sainte-Clotilde, La Réunion, France; 5 Polo d’Innovazione Genomica, Genetica e Biologia (PoloGGB), Via San Andrea delle Fratte, 06132 Perugia, Italy

**Keywords:** sperm selection, sperm competition, sterile males, SIT, stable isotopes

## Abstract

**Simple Summary:**

For the sterile insect technique to be successful, sterile male mosquitoes need to outcompete fertile males and mate with wild females. Some female insects, however, have been shown to be able to select for “better sperm” to secure high-quality offspring. This study used stable isotopes to mark the sperm of sterile and fertile males to find any evidence of female tiger mosquitoes selecting the fertile sperm over the sterile one when mated by both types of males. Although this study does not prove that a complex mechanism of sperm selection does not occur, we were not able to find any evidence that double-mated *Aedes albopictus* females favor fertile sperm for egg fertilization.

**Abstract:**

The key to success in the application of the sterile insect technique (SIT) relies on the ability of released, sterile males to outcompete their fertile wild male counterparts to mate with wild females. However, many insect species exhibit multiple-mating behavior, which can be a way for females to select paternity for their progeny. This study aims to recognize the consequences of potential double-matings during an SIT program and to detect any evidence of sperm selection favoring sperm from fertile mates. This report provides a descriptive analysis of the storage and use of sperm by female *Aedes albopictus.* Stable isotopes were used to mark the sperm of fertile and sterile males. Mated females were allowed to oviposit before dissecting the spermathecae to link the presence of each type of sperm to the sterility of the eggs laid. It was found that sperm in females inseminated by both males was distributed in the three spermathecae with no obvious pattern, mostly mixed but also separately, and no evidence of any mechanism for sperm selection, sperm precedence, or sperm competition in *Ae. albopictus* females could be found. The fact that only a few double-mated females were double-inseminated and could also produce semi-sterile eggs, together with the finding that the sperm of sterile males appeared to be no less viable than that of fertile males, is an encouraging outcome for SIT approaches.

## 1. Introduction

The success of the sterile insect technique (SIT) [1] against disease-transmitting mosquitoes relies on several key factors, such as the efficient mass-rearing of the target species, a reliable sex-separation method to remove potentially disease-transmitting females, effective reproductive sterilization, and high mating competitiveness of the sterile males once released. The efficiency of the SIT can be improved by enhancing the methodologies supporting all of these components, and achieving high biological quality of the sterile males is the key driver for this vector control tool. However, if the female counterparts in the wild are polyandrous and can preferentially select for the fertile sperm when double-mated by a fertile and sterile male, the success of the SIT would be compromised no matter how strong the technique’s components are.

Insects can exhibit different forms of remating patterns, which have fundamental consequences on sexual selection and sperm usage. Although multiple matings are accompanied by costs, such as time, energy (i.e., in finding a mate, courtship, and copulation), and risks of predation, multiple insemination can also have benefits, such as replenishing sperm supplies for egg fertilization and increasing the probability of mating with a high-quality mate, which also increases the probability of progeny producing high-quality sperm [2]. This, however, assumes the “sperm sexual selection hypothesis” [3], whereby the sperm can compete and/or the higher-quality sperm can be selected by the female. Such a phenomenon could have detrimental effects on the success of sterile insect techniques as the sterile spermatozoa might be counter-selected for egg fertilization. For some target insect species of the SIT, the occurrence of multiple mating has not been shown to affect the efficacy, as no sperm preference seems to occur, for example, in tsetse flies (*Glossina austeni*, *G. palpalis*, and *G. morsitans* [4]).

Fully inseminated females may mate with additional males if they have superior characteristics to the previous males [5]. Therefore, males can only control fertilization by evolving means, by which they can inhibit their female mates from remating, either by providing mating plugs or refractory pheromones. An extreme example by which males can control paternity can be observed in the damselfly, where males can remove sperm from previous mates from the female spermathecae [6].

Some insects, such as *Drosophila pseudoobscura*, are known to be highly polyandric, with 43% of a studied Mexican cohort carrying sperm from two or more males [7]. Numerous studies have been conducted to unravel the reproductive biology of Drosophila. Johnson and Zarrow [8] and Olivieri [9] concluded from their studies that there is sperm competition in females mated with multiple males. Childress and Hartl [10] reported that there is a sperm preference, i.e., preferential sperm storage, in females. Overall, the consensus has been that sperm usage for egg fertilization is non-random; however, the exact mechanism thereof is unknown, and seemingly highly complex [11].

Contrary to Drosophila (and Tephritidae), for which the literature is more abundant, there are still various gaps in the knowledge of remating in mosquito species. As the use of the SIT for managing mosquito species is moving towards the implementation phase [12], it is of high interest to understand the mechanisms of multiple insemination in females.

Mating behavior and physiology can vary greatly among different mosquito genera. *Culex pipiens* was found to be basically monandrous, although multiple insemination could occur within 48 h after the first mating [13], during which the formation of a mating plug from the first copulation allowed only 10% of eggs to be fertilized by a second male [13]. The production of an accessory-gland pheromone (with a delayed effect) then completely inhibited further insemination for the rest of the female’s life [13]. In *Aedes* mosquitoes, polyandry seems to occur at a substantial frequency in the field: 26% for *Ae. albopictus* wild females in La Reunion [14], 14% for *Ae. aegypti* females in large semi-field enclosures [15], and 6.25% (with an upper estimation limit of 14.6%) in wild female *Ae. aegypti* from Louisiana [16,17]. In species of the *Aedes* genus, mate rapprochement is undertaken by the males, either singly or as part of a small number of males flying in a swarm-like group [18,19]. *Aedes* males often show aggressive mate-seeking and mating behavior in both rearing cages and in the field, attempting to grab a female already *in copula* or sometimes even grabbing another male (Oliva, pers. observation); this behavior can easily increase the occurrence of multiple matings. These swarm-like groups have often been observed near bloodmeal hosts, in the shade of trees, or over potential oviposition sites [18], increasing the chances to encounter females, either virgin or already mated. Dieng et al. [20] observed under laboratory conditions that the presence of a host triggered the mating activity of males, while the mating activity was enhanced by the group effect when no bloodmeal host was available. They also showed that an already blood-fed female accepted copulation, although more reluctantly. Unfortunately, their study did not observe the actual insemination of the copulating females or record the duration of copulation, as there can be a high proportion of females accepting to copulate without a sperm transfer taking place (pseudocopulation), especially in shorter copulations in *Ae. albopictus* [21].

When inseminated by a male, female *Aedes* first store the semen in the *bursa inseminalis*, a pocket-like organ situated at the beginning of the gonotreme into which the male ejaculates (as reviewed in Oliva et al. [22]). This organ is connected to the spermathecal duct, and after a few minutes [21], the sperm cells migrate (actively or passively) to the spermathecal duct, which divides into three tubes connected to each one of the three capsules (spermathecae). In addition to sperm, male *Aedes* semen contains secretions from the male accessory gland (MAG). Sperm cells, and possibly non-sperm components (seminal fluid proteins (SFPs)) migrate to the spermathecae, while a significant proportion of the MAG secretion remains in the bursa, where it was observed to start to solidify after 30 to 40 min in *Ae. albopictus* [21]. Components from this secretion are known to inhibit further storage of sperm by providing a short-term physical barrier in both *Anopheles* [23,24], and *Aedes* species [25], and by a subsequent long-term chemical action by SFPs that can cause structural changes in the female reproductive tract [15]. The actions of MAG secretions and SFPs are not limited to the bursa, and are important in a range of longer-term female postmating responses, including remating, egg laying, flight, and even feeding behavior in many insects, thus inhibiting further mating with another male (reviewed in [24]), and their actions are complex and vary between species [26,27].

The filling mechanism of the spermathecae is still not entirely understood. In *Aedes* species, the third spermatheca is very rarely filled, although much sperm is still present in the *bursa inseminalis* [21,28]. The quantity of sperm stored by the female in the other two spermathecae, however, is largely sufficient for her to fertilize eggs over repeated gonotrophic cycles. Laboratory assays showed that multiple insemination of *Ae. albopictus* could occur only if the female was mated by different males within a 40 min interval; however, only 15% of the observed multiple copulations during this time window resulted in actual multiple insemination [21]. For these double-inseminated females, sperm originating from both males were used for egg fertilization at the first oviposition, but their use for the following gonotrophic cycles varied and could be dependent on the quantity of sperm transferred by each male and on the distribution of those sperm within the spermathecae [21]. Studies on *Ae. aegypti* showed that reinsemination was possible within a period of 2 h but no smaller intervals were tested; the persistence of a low rate of second-male semen transfer, even when the second copulation occurred between 2 and 22 h after the first one, was reported; however, it was not reported whether this semen was transferred to the spermathecae or only into the bursa [17]. A later study using the same strain, however, did show that the sperm transferred from two males was indeed stored in the spermathecae, as both males were found to sire the progeny [29].

In spite of its key role for mosquito reproductive success, no studies have yet focused on the process of egg fertilization within the female mosquitoes. Female insects generally fertilize the eggs one-by-one when they come to rest in the uterus, just before laying them [30]. Degner and Harrington [31] reported that in *Ae. aegypti*, sperm moves from the spermatheca down the spermathecal duct and is released one-by-one to enter the egg micropyle just before it is laid. The questions raised within this present work attempts to fill some of the gaps in the knowledge of mosquito sperm storage and egg fertilization reviewed by Oliva et al. [22] and Degner and Harrington [31]. We investigated the mechanisms of egg fertilization in *Aedes* females, observing the progeny of a female mated by both a sterile and a fertile male with different stable isotope markings. To detect any evidence of counterselection of sterile sperm, the distribution of paternity inside the spermathecae and in the progeny gives hints as to whether the sperm from one spermatheca at a time is used for egg fertilization, or if the sperm from the two or three spermathecae can be randomly used during the same oviposition event.

## 2. Materials and Methods

### 2.1. Rearing Procedures

The activities were conducted in part at Polo d’Innovazione Genomica, Genetica e Biologia (Polo GGB, Perugia, Italy) and at the Insect Pest Control Laboratory (IPCL), Joint FAO/IAEA Centre of Nuclear Techniques in Food and Agriculture, Seibersdorf, Austria.

The colony of *Ae. albopictus* used for the experiment originated from field collections in Rimini, Northern Italy, in 2004, and was maintained under laboratory conditions at the Centro Agricultura Ambiente, Bologna, Italy. The strain was transferred to the insectary of Polo GGB in 2014, and to the IPCL in 2012, where adults were kept in a climate-controlled room maintained at 27 ± 1 °C and 60 ± 10% relative humidity with a light regime of a LD 16:8 h photoperiod, including dusk (1 h) and dawn (1 h). Adults were kept in standard 30 × 30 × 30 cm cages (Megaview Science Education Services Co., Ltd., Taichung City, Taiwan) and continuously supplied with 10% (*w*/*v)* sucrose solution. For egg production, females were offered a bloodmeal on defibrinated bovine blood or fresh porcine blood using a Hemotek feeding apparatus with modified plates (Discovery Workshops, Accrington, Lancashire, UK) and were allowed to oviposit in plastic beakers containing deionized water and lined with coffee filter paper. Five days after the bloodmeal, the egg paper was removed from the cage and left to dry slowly at ambient conditions for three days. The eggs were kept in a closed container for at least one week before being used for hatching. Egg hatching was triggered using dehydrated rabbit food in deionized water. Larvae were reared at a density of approximately 500 first instar larvae (L1) per tray (30 × 40 cm) containing 1 L of water, and supplied an IAEA larval diet as described in the guidelines for routine colony maintenance of *Aedes* species [32]. Pupae were collected and placed in small plastic cups inside a clean adult cage for emergence.

### 2.2. Determination of Isotopic Marking Method, Rationale, and Establishment of Controls

To determine the isotopic marking methods and amount, spiking solution, and the cut-off values for the determination of positive or negative results, the following needed to be considered:

Both the sperm and the spermathecae in all tested samples are of unknown mass and comprise too little biological mass (and thus content of ^13^C and ^15^N) to be detected both on an analytical balance and by the isotope ratio mass spectrometry equipment. For this reason, the samples were spiked with a spiking solution of known ^13^C and ^15^N enrichment to bring the signal of the analyzed C and N isotopes to above the detection limit of the equipment. However, by adding the spike solution, the signature of the ^13^C and ^15^N were diluted in the sperm plus spermathecae samples. Therefore, high enrichment levels were needed to mark the sperm of the sterile and fertile males to obtain a reliable signal (delta values) for each stable isotope to determine positive or negative results for each sample. The delta values for each sample (each female/spermatheca) show a degree of variation, as sperm amount and spermathecae size are not the same in each individual female. Thus, the control samples and the treatment samples also show a range of delta values. Positive and negative control samples are plotted (together with the treatment samples) and cut-off limits for positive and negative results (presence of one or both types of sperm, yes/no) were established. Controls comprised the spermathecae of unmated females and females mated to unlabeled males only (negative controls), or spermathecae of females mated to labeled males only (positive control). Analytical (technical) repetitions are not possible, as each sample is incinerated in the analysis. Each female is considered a biological repetition.

A careful calculation of the natural contents of the 2 stable isotopes found in the IAEA standard larval diet and preliminary tests was performed to establish a reliable marking with either stable isotope.

Detailed materials and methods are described in the following sections.

### 2.3. Isotopic Marking

A preliminary validation of the isotopic marking protocols was made based on the methodology developed by Helinski et al., [33]; for both isotopes (^15^N and ^13^C), the target enrichment in the mosquito was 5 atom% (ref IAEA manual). The IAEA diet N content approximates 9.46%, and an enrichment of 20% ^15^N was chosen using glycine (98 atom%). The C content of the IAEA diet approximates 47.23% and the target enrichment was 25 atom%.

Eggs were hatched as above. The next-day 100 first instar larvae were counted and placed in a tray (20 × 30 cm) containing 175 mL of deionized water to which the IAEA larval diet was added. For the ^15^N-labeling tray, 12.9 mg of ^15^N-glycine (Cambridge Isotope Laboratories, Inc., Tewksbury, MA, USA) was added to the larval water on the first day only (Table 1), while, for the ^13^C-labeling tray, a solution of 147 mg of ^13^C-glucose (40% C by mass) diluted in 25 mL of water was used. The solution was added to the larval water gradually from day 1 to 5 (as detailed in Table 1) in order to prevent fungus development due to the high glucose content. The isotope solutions were added at the same moment of the diet, which was distributed as shown in Table 1.

Pupae were sexed under a stereomicroscope and, to guarantee accuracy of the sexing, were placed in small tubes for emergence to verify the sex once more. Adults were separated into different cages according to sex on the day of emergence and were continuously supplied with a 10% glucose solution (*w*/*v)*.

### 2.4. Experimental Set Up

#### 2.4.1. Storage of Multiple Sperm (Further Referred to as “Sperm Storage Experiment”)

Forty-three virgin unlabeled females (5–6 days old at first mating to ensure sexual maturity) were offered to mate with virgin males (5–10 days old, according to the day the remating occurred—see below) that were labeled either with ^15^N or ^13^C. One-to-one matings were performed to ensure the reliability of the observations; one female and one male (either ^15^N- or ^13^C-labeled) were released in a 30 × 30 × 30 cm cage. In total, 21 pairs were matched using ^13^C males as the first mate, and 22 pairs with ^15^N males as first male. An observer recorded the mating behavior and timing as rejection behavior by the female and none or several attempt(s) by the male, and copulation duration was also recorded.

The first male was removed immediately after copulation (copulation was verified with observation that the claspers of the males grabbed the female terminalia); only copulations longer than 20 s were considered successful, as based on previous studies [34]. A second male (with a different isotopic label) was immediately placed in the cage using a mouth aspirator and the delay between the two copulations and the duration of the second copulation were recorded. In most cases, successive attempts were made by the second male, which were either recorded as successful (>20 s) or unsuccessful.

In the case where the second male never succeeded to copulate within the first 20 min, the male was removed and the female was isolated in a tube with a cotton pad soaked in sugar solution until she was offered a new male again after 48 h. The delay of 48 h was selected, as by then, the bursa inseminalis was usually empty in all *Ae. albopictus* females. If no copulation happened after the 48 h, another trial was made at t + 72 h and t + 96 h.

**Figure 1:**
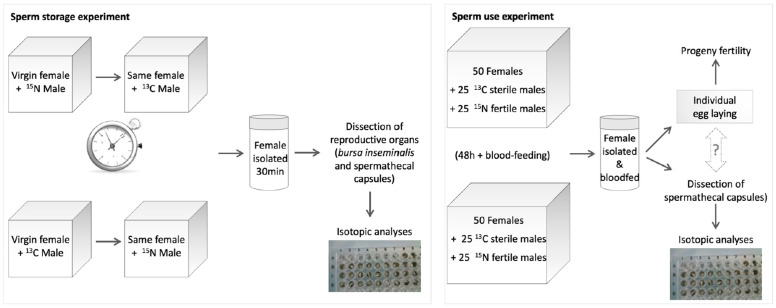
Experimental design.

After 30 min following their second copulation, the females were frozen and kept for later insemination status and labeling analysis (Figure 1).

#### 2.4.2. Use of Multiple Sperm Types for Egg Fertilization Following Double-Mating (Further Referred to as “Sperm Use Experiment”)

*Ae. albopictus* mosquitoes were reared and marked either with ^15^N or ^13^C, as described above. Pupae were sexed visually under a stereomicroscope. Half of the males marked with ^13^C and half of the males marked with ^15^N were irradiated with 40 Gy using the Gammacell220 of the IPC Laboratory. The dosimetry system used to verify the dose received by the batches was based on Gafchromic HD-V2 and MD-V3 film (Ashland Advanced Materials, Bridgewater NJ, USA) following the IAEA standard protocol for dosimetry [35]. Adults were caged separately according to sex, isotope label, and irradiation status, and were supplied with a 10% glucose solution (*w*/*v*).

Mating crosses were set up in bugdorm cages (30 × 30 × 30) on day 4 (after emergence) and left for 48 h, as follows:

- Cross 1: 25 × (^15^N sterile males) + 25 × (^13^C untreated males) + 50 × (unlabeled females)
- Cross 2: 25 × (^13^C sterile males) + 25 × (^15^N untreated males) + 50 × (unlabeled females)

Females were offered a bloodmeal (fresh porcine blood) on days 5 and 6. Engorged females were transferred to individual tubes for oviposition on day 7. After oviposition, females were frozen and kept for spermathecae dissection and analysis. Each female’s progeny was left to hatch in a tube of water and hatched and unhatched eggs were counted to calculate the fertility rate. Unhatched eggs were additionally bleached to verify fertility status [36].

Because of a low number of females laying substantial egg batches, all mating crosses were replicated three times. A total of 21 females laid eggs in the 3 repetitions in Cross 1 (where sterile males were marked with ^13^C), and 21 females in the 3 repetitions in Cross 2 (sterile males marked with ^15^N). Females not laying eggs were not checked for insemination status.

Females that laid eggs were analyzed individually according to the fertility rate of their progeny and the isotopic marking that was detected in their spermathecae (indicating which sperm was fathered by the sterile male.

Control fertility values were obtained from 6 crosses with fertile unlabeled males, and 6 crosses with 40-Gy-irradiated unlabeled males only (referred to as sterile males). It was shown previously that labeling with stable isotopes has no effect on the fertility of males [36].

### 2.5. Sample Preparation for Stable Isotopic Content Analysis

Females were dissected and their 4 storage organs were separated for isotopic analysis: bursa inseminalis (only for the sperm storage experiment), big spermatheca, and the two small spermathecae. For control individuals, empty organs from same-aged unmated females and females mated with unlabeled males were processed in the same manner.

Procedures for isotopic measurement followed a recommended methodology [30,32]. Each dissected organ was placed on a small piece of quartz paper in a tin cup; all tin cups were aligned in a 96-well plate and left to dry at 55 °C for several days. In order to increase the detection level of the isotopes contained in the small organs, a spiking solution was prepared using 2.00 µg N/µL of AS2 (ammonium sulfate, isotopic value delta ^15^N: 0.4‰) and 1.68 µg C/µL of SF1 (C3 sugar, isotopic value delta ^13^C: −26.07‰). A total of 5 µL was added into the tin capsules with the samples, which were then were dried at 40 °C, closed, and kept in the refrigerator until analysis.

The samples were analyzed for ^15^N and ^13^C content using an elemental analyzer (Vario Isotope Select, Elementar, Langenselbold, Germany) coupled to an isotope ratio mass spectrometer (Isoprime 100, Elementar).

### 2.6. Isotopic Data Analysis

The raw (measured) delta values were normalized to international scale by two-point calibration with reference materials and in-house laboratory standards (IAEA-N-1, IAEA-N-2, SF1, SS1). The total C and N content (µg) of the samples was calibrated against SS1 and IAEA-N-2, respectively [37]. A detailed description of the calculations on isotopic mass balance, enrichment levels, and mass of elemental carbon or nitrogen transferred to the females can be found in Supplementary File S1.

The estimation of the sperm amount here represents only an indicator of which type of sperm is more or less abundant and how that then reflects in the fertility of the female. Due to the high variations in the estimated amount of sperm both in single-mated females (from 45 pg to 1350 pg of estimated sperm amount; average + stdev = 519 ± 366) and double-mated females (from 195 pg to 1385 pg of estimated sperm amount; average + stdev = 609 ± 309), we did not use these data for statistical analysis. In addition, the amount of sperm (or male ejaculate) transferred to the spermathecae estimated here may not be representative of actual sperm abundance, as the experimental design cannot differentiate marked sperm from marked accessory gland-derived seminal fluid. Additionally, a large proportion of the sperm transferred by males remains in the bursa, which could not be analyzed, as the bursa content was dissolved within 24–48 h, and therefore before egg laying. The description of the data here is, therefore, purposely more qualitative than quantitative, with the goal of understanding whether specific patterns of sperm storage and sperm could be detected.

### 2.7. Comparison Analysis for the Sperm Use Experiment

The females were grouped according to the sperm distribution in their 3 spermathecae and the fertility value of their progeny. The reference values from single mated females were as below:

- HRS max = 3.3%, HRS average = 0.5 ± 1% for females inseminated by sterile males only
- HRF min = 87.5%, HRF average = 96 ± 3% for females inseminated by fertile males only

The threshold value for “semi-sterility” was calculated as HRS max + 2 × standard deviation. The semi-sterility threshold was then a 5.3% egg hatch rate.

The threshold values for “semi-fertility” were calculated as HRF min − 2 × standard deviation. The semi-fertility threshold was then 79.3%.

The individual females were therefore grouped as follows:

Group A “Fertile eggs only”: Both sperm present in the spermathecae and laid eggs hatching ≥ 81%.

Group B “Sterile eggs only”: Both sperm present in the spermathecae and laid eggs hatching ≤ 4.8%.

Group C “Semi-fertile eggs”: Both sperm present in the spermathecae and laid eggs hatching ranging 50.1–81%.

Group D “Semi-sterile eggs”: Both sperm present in the spermathecae and laid eggs hatching ranging 4.8–50%.

### 2.8. Statistical Analyses

Bartlett’s test indicated homogeneity of variance (*p*-value = 0.5) for the copulation duration variable, although it did not follow a normal distribution. A Pearson correlation test was used to test for correlation between the copulation duration and the number of spermathecae filled for the sperm storage experiment.

Due to difficulties in obtaining large numbers of females remating and laying eggs with such “contrived” mating experiments in mosquitoes, the data obtained are not fit for statistical analyses but remain very informative of the variety of the potential patterns of storage and use, allowing conclusions to be drawn about mating dynamics in *Aedes albopictus*, in terms of the presence or absence of preferential sperm use or sperm competition.

## 3. Results

### 3.1. Relation between Insemination and Copulation Duration

Of the 43 first copulations observed in the sperm storage experiment, 4 did not result in an insemination (Table 2), although those pairs appeared to be copulating for 18 s (2 pairs), 27 s, and 5 min. All 39 of the copulations leading to a single successful semen transfer lasted between 22 and 121 s, with a median duration of 39 s. Among these single-inseminated females, 39%, 55%, and 6% had 1, 2, and 3 spermathecal capsules filled, respectively.

There was no statistical correlation between the number of spermathecae filled and the copulation duration of the first (successful) mating for single-inseminated females (Pearson correlation test, t = 0.8, df = 32, *p* = 0.4). The two females showing the three spermathecae filled were only inseminated by the first male and their copulation had lasted 35 s and 45 s.

### 3.2. Immediate Double Copulations

In this sperm storage experiment, the second male generally had difficulties in successfully grabbing the female; however, if it succeeded, then it was the male that ended the copulation. When the second male would fail its first copulation attempt, the female usually then kept rejecting its subsequent attempts. In total, 27 females (out of 43) accepted to remate immediately after their first copulation (Table 2), in which the duration of the second copulations ranged from 12 s to 5 min05 (with a median of 24 s), and 41% of the males attempted two copulations of over 12 s. Of these, only four twice-copulated females were actually inseminated by both males (i.e., 15% of the immediate double copulations, or 9% of all tested females). The delay between the two (successful) inseminations varied between 1 min37 and 4 min53 (Table 2). The intercopulation delay for the other females ranged from 49 s to 14 min, with a median of 2 min 20 s; however, it must be noted that the observations were interrupted if no second copulation occurred after 20 min.

Table 3 describes the sperm distribution for the double-inseminated females. For three females, the sperm from both males were mixed in the bursa and in the big spermatheca. Of these, only two had sperm in one of the small capsules, and it came from the second male. The fourth female stored only the sperm from the first male in the big and one of the small spermatheca, although the bursa contained semen from both, and the intercopulation period was the shortest.

### 3.3. Late Second Copulations

When the second male was presented 48 h after the first copulation, the female was generally reluctant to copulate, while the male made several attempts but ended them after a few seconds. Out of the 16 females remaining (which did not accept an immediate remating), 9 accepted a second copulation (>10 s) after 48 h, 5 after 72 h, and 2 after 96 h (females were removed after second copulation) (Table 2). The median duration of this second copulation was 16 s, with a few very long copulations (200 and 700 s); half of the males made successive attempts to copulate.

The isotopic analysis of the bursa inseminalis indicated no semen content in most of these females. However, traces of the first male’s semen were detected in two females frozen 48 h after the first mating and four females frozen 72 h after.

Only one female received semen from the second male 72 h after the first insemination, but this semen was contained only in the bursa and was not transferred to the spermathecae; only the first male’s sperm was present in the big spermatheca and one of the small ones.

### 3.4. Dosimetry of Sterile and Fertile Controls

The dosimetry confirmed that all doses received laid within a 3.07% error range.

Eggs laid by females mated with *Ae. albopictus* males irradiated with 40 Gy had a hatch rate of max = 3.3%, average = 0.5 ± 1%, plus 2 × standard deviation, and the egg hatch rates of the fertile controls were min = 87.5%, average = 96 ± 3%, minus 2 × standard deviation.

### 3.5. Relationship between Sperm Distribution and Fertility 

During the sperm use experiment, only 42 females laid substantial egg batches out of the 300 that were isolated after the 48 h mating period, across the six repetitions (three repetitions for the two crosses). Females laid an average of 63 ± 25 (sd) eggs. The often low number of eggs laid could be due to the skip-oviposition behavior of *Ae. albopictus*, and the small tubes with clean water may not have been a preferred oviposition environment.

Of those 42 females, 21 showed dual-labeling, indicating double insemination by different types of males. Unfortunately, no data are available in this experimental design on the duration of mating(s) for each female, or on the occurrence of multiple mating from the same category of males (i.e., either sterile or fertile).

Overall, 79% of the ovipositing females had two spermathecae filled, while 13% had three filled.

**Figure 2:**
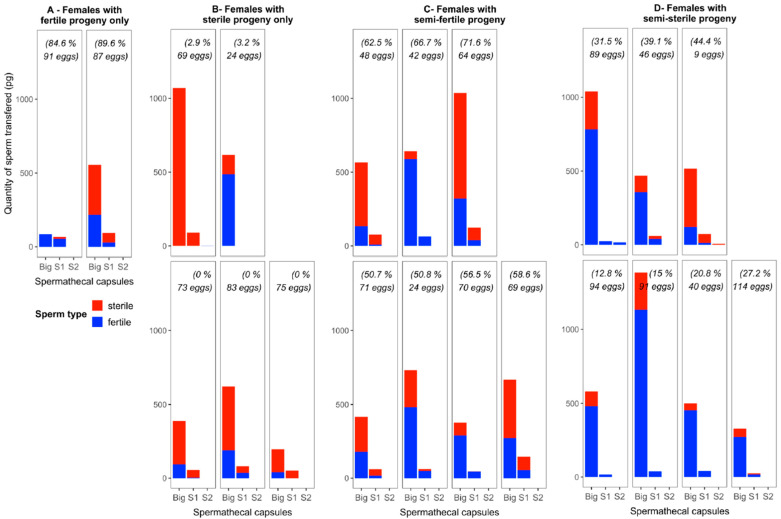
Estimated quantity (pg) of sperm type distributed in each female spermatheca and level of egg fertility in females where two isotopic levels were detected (double-labeled females). Females are grouped according to the level of fertility of the progeny: only fertile (**A**), only sterile (**B**), semi-fertile (**C**), and semi-sterile (**D**). The estimated quantity of sterile or fertile sperm is showed for the three spermathecae of each female: “Big” as big capsule, “S1” as first small capsule, “S2” as second small capsule (there is no order in the small capsules dissected; the one listed as “first” is the one with more sperm content). The percentage value within each box indicates the fertility value of each female’s progeny during the first oviposition event; the total number of eggs laid is indicated.

Figure 2 shows the distribution of sperm type in each spermathecal organ for females where the two isotopic labels were detected. In most females, the two labels were mixed; 57% of females stored the two labels in two spermathecae. In 38% of females, two labels were present in the big spermatheca only. Where a label was detected in the small spermatheca(e), it was always the label of the most abundantly present sperm type found in the big spermatheca. One female, however, showed a mix only in one small spermatheca. Lastly, only one female showed the two labels stored in a different organ; however, there was only a little amount of the ^13^C label (from the fertile male) in its third spermatheca, while the ^15^N label was abundant in the big and second spermatheca.

The females in Figure 2 are grouped according to their resulting egg fertility. Two females laid only fertile eggs (Group A, 84.6 and 89.6% of egg hatching) even if their spermathecae also stored sterile sperm; for one of these females, the estimated amount of sterile sperm (or total ejaculate) even appears as more abundant.

On the other hand, five females laid sterile eggs (Group B, 0–3.2% of egg hatching), although their spermathecae also contained fertile sperm. Three of those females showed more abundant estimated amounts of sterile sperm than fertile sperm in both or only the big spermatheca(e). However, for one female, with only the big spermatheca containing sperm, the fertile sperm was over three times more abundant than the sterile sperm; however, the fertility rate of the eggs was 3.2%. The last female of this group stored only a little amount of fertile sperm in the third spermatheca, but it was not mixed in the other two organs.

In the group of females laying semi-fertile eggs (50.7–71.6% of egg hatching), both types of sperm were mixed in the two filled spermathecae. Four out of these seven females had more abundant sterile sperm in these two spermathecae. In the group of seven females laying semi-sterile eggs (12.8–44.4% of egg hatching), only one female had more sterile sperm stored in the two spermathecae and still showed 44% hatching eggs. For the three females from this group with the lowest fertility (12.8, 15, 20.8%), the sterile sperm was detected only in the big spermathecae, and in small estimated amounts as compared to fertile sperm.

## 4. Discussion

The observations in this study have brought an interesting light on the reproductive biology of *Ae. albopictus*. This study showed that small proportions of females can be inseminated by different males, and when this happens, both the sperm storage and sperm use for egg fertilization appear to have no readily distinguishable pattern.

### 4.1. Multiple Insemination in Aedes albopictus Is Not a Common Phenomenon

The duration of successful copulations had a median of 39 s, corroborating with what has already been reported for *Ae. albopictus* [21], and was longer than the reported average duration for *Ae. aegypti* [28,38,39]. The proportion of females with two spermathecae filled was 47% when one-to-one matings were performed, with no correlation to the duration of mating or occurrence of double-matings. This proportion increased to 79% when groups of females were caged with groups of males (density of 3704 mosquito/m^3^). This reflects what was reported other studies, suggesting that males may generally transfer enough sperm to fill only one spermatheca. It seems that the presence of multiple males in a cage may trigger each male to transfer more sperm during the copulation, therefore leading to a higher proportion of females with two spermathecae filled. Another explanation could be that most females with two spermathecae filled have been mated by more than one male [14,40]. This highlights the need to carefully select the mosquito density when studying mating and competitiveness in cages, as it may easily not reflect natural behavior in the wild.

We report only 9% successful reinsemination during one-to-one successive matings (regardless of the delay between the copulations); this is the same rate reported in an earlier study using similar crosses on this species [21]. Under colonization, in small rearing cages where the chances of male–female encounters are forced, the rate of multiple insemination can increase, as indicated by the usual proportion of intermediate fertility values from competitiveness studies where fertile and sterile males are competing in relatively high densities [41,42,43]. This is corroborated by the higher proportions of multiple-inseminated females in our second experiment: when groups of males and females were caged, dual-labeling was detected for 50% of the females laying eggs, although this does not indicate the occurrence of multiple same-label matings.

The delay between the two copulations that led to successful second insemination was less than 5 min, as reported in Oliva et al. [21]. However, one female showed the presence of sperm from the second male in the *bursa inseminalis* for a mating occurring 72 h after the first one, but this sperm was not transferred to the spermathecae, suggesting either a chemical blockage (unlikely), or the females’ unwillingness to store additional sperm. Only sperm transferred to the spermathecae will be able to fertilize eggs, as the content of the bursa is progressively dissolved. Early reports by Spielman [25], Gwadz [44,45], and Craig [46] describe that females are involved in the sperm storage process. Noble et al. [47] show that sperm is modified after storage, and that there may be a window in which females can store sperm, after which she no longer accepts new sperm. The seminal fluids transferred by the male during insemination are reported to trigger a phenomenon of refractoriness to successive insemination [21]. Degner and Harrington [17] reported higher rates and a longer period of reinsemination acceptance for female *Ae. aegypti*; however, they considered the females as polyandrous when they had received a second semen into their bursa, regardless of whether this sperm reached the spermathecae, while Ramirez-Sanchez reports that, indeed, polyandrous females can use both sperm to sire their progeny [29].

Female *Ae. albopictus* do not seem to actively seek a second insemination, as they often showed a rejection behavior. Moreover, the second insemination was not correlated to a better filling of the spermathecae, as the second small one remained empty in most of the cases, and the first small one was not always filled. As non-useful copulations could lead to a decreased fitness for females or subjection to increased predation risks in the field [2], some mosquito species seem to have adapted behaviors that allow females to avoid successive copulations. If this behavior has not been counter-selected in *Aedes* species, with neither females nor males being able to detect for mating status before copulation, then the cost of multiple copulations is likely to outweigh the benefits of multiple insemination.

### 4.2. No Obvious Pattern of Sperm Storage and Use after Multiple Insemination

Females of most *Culicinae* species possess three spermathecae [48], which makes them an interesting model to study differential sperm storage and sperm use following multiple inseminations. Here, we report only one case of a separate distribution of both males’ semen between the spermathecae; a mix of the two sperm types could be detected in two spermathecae for 57% of the females. We report no clear pattern for sperm use in egg fertilization. It does not appear to be dependent on the estimated quantity, as a higher estimated quantity of sperm (and male ejaculate overall) transferred did not always secure the fertilization of the progeny for this male. Egg fertilization was also not clearly associated with the sperm distribution in the big or small spermathecae either. However, the data from females laying semi-sterile or semi-fertile eggs tend to indicate that the mix of sperm came from either only the big spermatheca or a mix of content from the big and small capsules. Although it could not be experimentally shown which of the two scenarios was occurring, our data show that the sperm used is unlikely to have come from the small spermathecae alone.

An important finding is that 33% of the females showing dual-labeling produced progeny belonging to only one of the two males. This sperm usage might vary for the successive oviposition, which may suggest some mechanism of sperm discrimination or pattern of use, but this could not be investigated here, as the experimental design required the killing of the female to analyze the spermathecae content. This finding highlights the limit of investigating polyandry by observing only the progeny, especially from only one oviposition event, as it may not reflect the actual polyandrous state of the female as shown by the distribution of sperm within the spermathecae. Thus, the previous studies using microsatellites on a few progeny [26,30] are most likely underestimating the actual rate of polyandry in the field.

The mechanisms of sperm usage have been investigated and described in other diptera, (e.g., *Scathophaga stercoraria* [49] and *Dryomyza anilis* [34]). These insects have morphological similarities to mosquitoes in terms of their spermathecae, thus offering some clues to the mechanisms of sperm storage and usage [31]. In the yellow dung fly *S. stercoraria*, sperm storage appears to be non-random, and the selection for egg fertilization is somehow influenced by a combination of the displacement of sperm present by the sperm of the last-mated male, and a mechanism by which the female can control sperm release, resulting in a “mate now, choose later” strategy [49] which was what was observed here. In *D. anilis*, the different sperm storage organs seem to have different functions when storing and using sperm, and males tap the females during mating to influence the sperm distribution patterns in the female [34]. In some insect species, the sperm usage pattern can be different to the pattern in sperm storage [34]. In females of other species (such as *D. melanogaster*), it was suggested that the female can even select sperm to favor the sex of the offspring [50]; however, this study dates to the 1970s and no reports could be found since that corroborate or refute this claim.

In the coleoptera *Tribolium castaneum*, offspring were mainly derived from the last male mated with, with progressive usage of sperm from previous males only when the sperm of the latest male became depleted [51]. The last-mating male also predominantly fathers offspring in some locust species (*Paratettix texanus*, *Schistocerca gregaria*) [51] and some Lepidoptera (*Trichoplusia* and *Papilio* sp.) [51]. We showed that *Ae. albopictus* females can either use only one sperm or a mix of sperm from two males, apparently without a distinguishable pattern. This has also been observed in fruit flies, for example, in *Trypetidae*, where sperm from two mates are used almost equally [51], unless oviposition occurs between matings, in which case the second male fathers the majority of the offspring (*C. capitata*, Caceres C., unpublished data). This phenomenon appears unlikely for *Ae. Albopictus*, as our current data and previous study [21] indicate that any successive matings occurring after an oviposition cannot lead to sperm transfer to the spermathecae.

### 4.3. Sterile Sperm Is as Competitive as Fertile Sperm

In this small study, no evidence for the counter-selection of sterile sperm for egg fertilization was found. In some instances, only a small estimated quantity of sterile sperm was enough to induce full sterility or fertility levels below 30%. The occurrence of multiple matings could have negative effects on the efficiency of an SIT approach if the sterile sperm is less competitive due to the ionization effect, or if the female would be able to differentially use it [52,53]. This study brings important knowledge showing that sterile sperm is able to secure egg fertilization even in the case of multiple inseminations and even if transferred in lower quantities. The lack of correlation between apparent sperm use and sperm storage warrants further investigation into the possibility of sperm choice.

It has been reported that a sterile male has a limited sperm capacity due to the impossibility to produce and mature new sperm cells (because of ionization damages), and thus has a limited number of potential mates that it is able to inseminate (on average five females for *Ae. albopictus* [21]). The outcome of this study will therefore be of high importance for the projection of *Aedes* SIT models, which should take into account similar chances for egg sterilization from both types of sperm [54,55,56].

It would be of interest to further study how sperm use can vary over several gonotrophic cycles; although there are experimental limitations to destructive approaches based on isotopic analyses of spermathecae. Although not presented in this study due to limited replications, preliminary results in *Ae. aegypti* using the same experimental method showed a similar trend in sperm storage and use as in its sister species without a detectable pattern.

### 4.4. Limitations of the Study

An important limitation of this study is that we did not intentionally vary the quality or genetics of the males used for the mating studies, which would presumably be the basis for cryptic female sperm choice. Furthermore, there was at least one individual that did have segregated first-male and second-male sperm in the spermathecae, which presumably would be a prerequisite for discrimination. There were also some anomalous cases where females with stored sterile ejaculate were nearly fully fertile, and conversely cases where mostly sterile females seemed to have stored abundant fertile ejaculate. Therefore, this may be at least a small indication that more may be at play in sperm use and discrimination than is readily apparent. To better observe any possible patterns, a much larger sample size of double-inseminated females would be needed, and more than one oviposition cycle examined. Another important factor to consider in future studies is the age of the males, as older males have an increasing amount of sperm, which may be an important factor affecting the amount of sperm transferred and could impact sperm competition outcomes [57]. However, for this current study, the aim was to observe whether the proportion of fertile eggs is somehow linked to the amount detected in the different spermathecae, and whether any evidence of sperm preference or use pattern for fertilizing eggs could be detected.

## 5. Conclusions

It is important for the SIT, where releases ensure overflooding ratios of sterile to fertile males, to maximize the chances of wild females encountering, and mating with, sterile males first. But if females were to be mated by both sterile and fertile males within a short timeframe, double insemination would still lead to the decreased fertility of the eggs, and the efforts of the sterile mate would not be futile. It is also reassuring from an SIT point of view that although more than half of the tested females accepted a second mate, only four of these were actually double-inseminated. We expect that in field conditions, these numbers are likely to be far lower than in the crowded cage environment. This study dismisses concerns of sperm competition favoring fertile sperm once stored in the spermathecae, as well as the displacement of the sterile sperm by fertile mates, and thus presents a positive finding for the SIT against the Asian tiger mosquito *Ae. albopictus*, and most likely *Aedes aegypti*.

## Figures and Tables

**Table 1:** Distribution of the amount of diet and isotope labeling.

	Day 1 (1st Instars)	Day 2	Day 3	Day 4	Day 5
IAEA diet	0.06 g	0.06 g	0.12 g	0.12 g	0.12 g
^13^C-glucose	1 mL	-	5.2 mL	6.3 mL	12.5 mL
^15^N-glycine	12.9 mg	-	-	-	-

**Table 2:** Number of females accepting a first and second copulation and resulting insemination status.

	Total Copulations Observed on the First Mating *	Number of Effective Inseminations of This First Mating Event	Number of Females Accepting a Second Copulation Act *	Number of Females Showing Single Insemination	Number of Females Showing Double Insemination
Total	43	39	43	39	4
Immediate second copulation	/	/	27	23	4
2nd copulation after 48 h	/	/	9	9	0
2nd copulation after 72 h	/	/	5	5	0
2nd copulation after 96 h	/	/	2	2	0

* Copulation observed for over 20 s.

**Table 3:** Sperm distribution in double-inseminated females. Duration of the two copulations and storage of semen from both males in the sperm storage organs (the other small spermatheca was never filled). Presence of semen by first and/or second male (identified by the isotopic labeling) is indicated by a cross.

1st Male Copulation (s)	Inter Copulation Delay (s)	2nd Male Copulation (s)	Bursa Inseminalis		Big Spermath.		1st Small Spermath.	
			1st Semen	2nd Semen	1st Semen	2nd Semen	1st Semen	2nd Semen
22	353	305	X	X	X	X		
26	107	31	X	X	X	X		X
40	179	71	X	X	X	X		X
28	97	148	X	X	X		X	

## Data Availability

The datasets used and/or analyzed during the current study, including all dosimetry reports, are available from the corresponding author upon reasonable request.

## Supplementary Materials

The following supporting information can be downloaded at https://www.mdpi.com/article/10.3390/insects15090721/s1, Supplementary File S1 “Isotopic data analysis”.
