# Supplementary material for: Sperm Storage and Use Following Multiple Insemination in Aedes albopictus: Encouraging Insights for the Sterile Insect Technique
Source: Insects. 2024 Sep 20;15(9):721. doi: 10.3390/insects15090721 (PMC11432246; doi:10.3390/insects15090721)
Supplement: Supplementary file 1 [file insects-15-00721-s001.zip › insects-3202771-supplementary.pdf]

Supplementary file S1:

Materials and Methods

Isotopic data analysis

The raw (measured) delta values were normalized to international scale by two-point calibration with reference materials and in-house laboratory standards (IAEA-N-1, IAEA-N-2, SF1, SS1). The total C and N content (μg) of the samples was calibrated against SS1 and IAEA-N-2 respectively (IAEA 2009).

Natural abundance values were determined by analysis of the unlabelled whole mosquitoes and or organs. Due to the low weight of the spermathecae a spike of N and C was added to each sample to attain sufficient quantity of either element to have enough signal for reliable isotopic analysis (Helinski et al. 2008). The atom% ^{13}C and ^{15}N was calculated for each spermatheca sample as $atom\% = \frac{100*Rr*(\delta/1000+1)}{1+Rr*(\delta/1000+1)}$, where Rr is the isotopic ratio of the reference standard.

<u>Sperm amount estimation</u>. Initially spermathecae elemental carbon mass was determined by isotope dilution of control mated females, specific for both big and small spermathecae. The size specific spermathecae sample mass was assumed across all samples of the same species, as it would have been impossible to reliably determine this value on an individual basis. Elemental N values were assumed to be one tenth of the carbon values based on insect stoichiometry (IAEA 2009). After accounting for the elemental specific spike, by simple subtraction. The proportion of the sample derived from the labelled sperm was calculated, again by isotopic mass balance, using a standard source product calculation, where enrichment of the product is divided through by the enrichment of the source. The source enrichment was assumed to be the isotopic enrichment of whole labelled male mosquitos, and whole body and sperm values were assumed to be the same value, no organ specific isotope

discrimination was assumed as these were labelling studies. Moreover, the same sperm enrichment value was assumed for all tests. Conservative positive mating threshold values (i.e. clear evidence of mating) were established based significant deviation (2SD) from the variation in the isotopic values of the virgin un-inseminated females, yielding a threshold value of approximately 5‰ difference. The mass of elemental carbon or nitrogen transferred to the females was calculated by multiplying the proportion value, by the assumed mass of the size specific spermathecae. This allowed us to attain reliable relative values of mass of sperm transfer across treatments.
